# Combined metagenomic and metabolomic analyses reveal gut microbiota dysbiosis and metabolic dysfunction in pediatric neurodevelopmental disorders

**DOI:** 10.3389/fimmu.2025.1645137

**Published:** 2025-09-02

**Authors:** Qi Wang, Yuanhao Luo, Chunmei Mao, Xuesong Xiang, Juanjuan Chen

**Affiliations:** ^1^ Cuiying Biomedical Research Center, The Second Hospital & Clinical Medical School, Lanzhou University, Lanzhou, Gansu, China; ^2^ The Second School of Clinical Medicine, Lanzhou University, Lanzhou, Gansu, China; ^3^ Department of Pediatric Rehabilitation, Gansu Province Hospital Rehabilitation Center, Lanzhou, Gansu, China; ^4^ Nation Health Commission Key Laboratory of Public Nutrition and Health, National Institute for Nutrition and Health, Chinese Center for Disease Control and Prevention, Beijing, China

**Keywords:** pediatric neurodevelopmental disorders, combined metagenomic and metabolomic analyses, gut microbiota dysbiosis, disturbed amino acids metabolism, decreased protein digestion and absorption, increased fat digestion and absorption, reduced purine and pyrimidine metabolism

## Abstract

**Introduction:**

Neurodevelopmental disorders (NDDs) are chronic brain diseases linked to innate immune signaling abnormalities, affecting children with complex gut-brain axis etiologies and limited targeted therapies. While infant microbes/metabolites may predict childhood NDDs, their landscape and host-metabolism interactions in NDDchildren remain unclear.

**Methods:**

This study enrolled 40 NDDchildren (mean age: 5.18 ± 1.77, F:M = 11:29) and 60 healthy controls (HCs; mean age:5.11 ± 1.42, F:M = 25:35) from Gansu Province Hospital Rehabilitation Center. Shotgun metagenomics and untargeted metabolomics was used to analyze gut microbiota and fecal/plasma metabolites, multi-omics integration analysis was performed to explore host-microbe interactions.

**Results:**

Clinically, NDD children showed self-care, concentration, and social behavior deficits, with grandparents as primary caregivers, versus parents in HCs. Microbiome analysis revealed reduced gut diversity and dysregulation in NDDs: depleted beneficial taxa including Akkermansia muciniphila and Lactococcus lactis, but enriched GABA/lactateproducing bacteria; and disrupted pathways included polysaccharides/fatty acids/amino acid/purine ribonucleosides metabolism. Fecal metabolomics identified 100 enriched metabolites including polyamines and GABA in 45 pathways and 254 depleted metabolites including bile acids and butyrate in 57 pathways. Plasma metabolomics showed 321 enriched metabolites like free fatty acids in 143 pathways and 270 depleted metabolites including glycerophospholipids in 84 pathways. Notably, phenolic acids, arginine/proline metabolism, and HIF-1 signaling were enriched in both feces and plasma of NDDs children. Benzene derivatives, indoles, steroid hormone biosynthesis, and tryptophan/tyrosine/phenylalanine metabolism were increased in plasma but decreased in feces, while oxidized lipids, amino acids and derivatives, metabolism of glycine/serine/threonine, alanine/aspartate/glutamate, and cysteine/methionine showed the opposite pattern. Venn analysis identified 29 common metabolites, with eight in KEGG maps. 11-dehydrocorticosterone, LPC (17:0/0:0), adipic acid, and sucralose were decreased in feces but increased in plasma; 1-methylhistidine and trigonelline were decreased in both; L-asparagine anhydrous was increased in feces but decreased in plasma; and sarcosine increased in both. Microbe-metabolite correlation analyses linked these metabolites to NDDs depleted species A. muciniphila, L. lactis, A. butyriciproducens, and etc.

**Discussions:**

Collectively, our study presents the first integrated profile of gut microbiome, microbial metabolites, and host metabolome, reveals gut microbiota dysbiosis, functional impairment, and metabolic disturbance in pediatric NDDs. These findings provide a theoretical foundation for microbiotaand metabolite-targeted therapeutic strategies in childhood NDDs.

## Introduction

Childhood neurodevelopmental disorders (NDDs), including autism spectrum disorders (ASD), attention deficit hyperactivity disorder (ADHD), disorders of intellectual development, and speech/language/learning disorders, are chronic brain conditions that severely impair children’s cognitive, behavioral, and social abilities (1, 2). With rising prevalence, NDDs impose significant economic and emotional burdens on families and healthcare systems (3). Despite extensive research on genetic, environmental, and neurobiological factors, the complex pathogenesis of NDDs remains incompletely understood (4).

Emerging evidence highlights the pivotal role of the gut microbiota in host neurodevelopment and behavior regulation (5). Through the bidirectional “gut-brain axis”—mediated by neural, immune, endocrine, and metabolic pathways—gut microbes influence brain function via multiple mechanisms (5). They produce bioactive metabolites such as short-chain fatty acids (6), neurotransmitters (7), and amino acid derivatives (8), directly modulating neural development. Additionally, they indirectly impact neurodevelopment by regulating intestinal barrier integrity, immune responses, and inflammation (9).

Recent studies have identified distinct gut microbiota alterations in NDDs. For example, ASD patients exhibit dysbiosis characterized by imbalanced *Bacteroidetes*/*Firmicutes* ratios and reduced short-chain fatty acid-producing bacteria (10, 11). Metabolomic analyses also reveal dysregulations in amino acid, energy, and neurotransmitter metabolism in NDD populations (12). However, these investigations predominantly focus on either microbiota or metabolome independently, lacking integrated approaches to elucidate microbiota-host metabolic interactions and their pathogenetic roles. Although a longitudinal study demonstrated the predictive potential of combined metagenomic and metabolomic profiling for early NDD risk assessment (13), the specific signatures of gut microbiota, metabolites, and microbial-host co-metabolites in childhood NDDs remain uncharacterized.

This study employs integrated metagenomic and metabolomic approaches to comprehensively profile gut microbiota and metabolites in children with NDDs and healthy controls (HCs). The primary objectives are to: (1) identify NDD-associated microbial taxa by comparing microbiota composition and function; (2) discover potential metabolic biomarkers and dysregulated pathways; (3) elucidate the roles of microbial-host co-metabolites in NDD pathogenesis; and (4) establish correlations with clinical phenotypes to inform early diagnosis and precision interventions. By advancing understanding of microbiota-metabolite-host interactions, this research will provide scientific underpinnings for developing microbiota-based therapeutic strategies, including probiotics, prebiotics, and metabolic modulation.

## Methods

### The aim, design and setting of the study

The present study was conducted to investigate the gut microbiota and metabolomic characteristics in children with NDDs. 40 NDDs children and 60 HCs were recruited from the pediatric rehabilitation department at Gansu Province Hospital Rehabilitation Center (Lanzhou, Gansu) according to the inclusion and exclusion criteria, which was shown as follows:

(1) Children presenting with symptoms of NDDs, such as developmental delays in motor skills, including sitting up, rolling over, crawling, and walking; communication difficulties such as delayed speech or impaired language abilities; slower mastery of self-care tasks (e.g., potty training, dressing, and feeding), limited memory capacity for quick learning, inability to comprehend cause-and-effect relationships, and challenges in logical reasoning and problem-solving abilities; (2) Participants aged between 3 and 12 years; (3) Undergoing standardized assessments for IQ (less than 70), cognition, communication, etc., provided by the hospital; (4) Absence of known inflammatory or chronic infectious diseases; (5) No usage of medications such as antibiotics that affect the gut microbial composition within the past month; (6) No comorbidity with other mental disorders (e.g., epilepsy, schizophrenia) to avoid their interference with gut microbiota and metabolites; (7) No consumption of fermented foods including yogurt and pickles in the last month; (8) Weaned participants with a diet primarily consisting of rice, cooked wheat products, meat, vegetables; (9) Daily milk intake less than 300 mL; (10) Complete availability of sample information and phenotypic data.

### The characteristics of participants, phenotypes and samples collection

Assessment scales, including IQ, Developmental Milestones for Infant Gross Motor Development, Fine Motor Function Development in Infants and Toddlers, Cognitive Function Development, Social Interaction and Emotional Development, Speech and Language Function Development, as well as Game Function Development were administered with the assistance of guardians for each child. Additionally, sex, age, height, weight, body mass index (BMI), premature or not, delivery mode, birth weight and height, age of parents at birth, guardians, self-care ability of daily living including eat independently, concentrate on the meal, picky, wash before meals, tidy, labor, chosen clothes, chosen toys, group activity, seek help were also recorded according to our pre-designed questionnaire.

The study met cross-sectional sample size requirements, with the control group sized at 1.5 times the study group. Sample size was calculated using the formula:


$$n\;=\;\frac{Z_{a/2}^{2}\cdot P\cdot (1-P)}{E^{2}}$$

With a 95% confidence level (Z_α/2_ = 1.96), NDD prevalence (P=1%-3% per WHO), and margin of error (E=0.05), minimum sample sizes were 8–23 for NDD cases and 12–35 for controls. The final sample included 40 NDD children and 60 healthy controls (HCs).

The stool samples were collected from each child with the assistance of their guardians and stored at -80°C. The blood samples were obtained by a professional nurse from the pediatric rehabilitation department of the hospital and immediately centrifuged to obtain plasma (4°C, 3000 rpm, 10 minutes) to store at -80°C. After all samples were collected, both fecal and plasma samples were transferred to the laboratory by dry ice for shotgun metagenomic sequencing and untargeted metabolomics analysis.

### Shotgun metagenomic sequencing

#### DNA extraction, library construction, metagenomic sequencing

Fecal samples were transported to the laboratory by dry ice to facilitate DNA extraction. The total DNA in each sample was extracted following a previously described protocol (37). The quantity and quality of the total DNA were assessed through the NanoDrop Spectrophotometer ND-1000 (Thermo Fisher Scientific). Metagenomic libraries were constructed using the TruSeq DNA PCR-Free Library Preparation Kit (Illumina), and their concentrations were determined by Qubit 2.0 fluorimeter (Invitrogen). Sequencing of the metagenomic libraries was performed using BGI-Seq500 with 150 bp paired-end sequencing of ~ 350 bp inserts at BGI-Shenzhen (Shenzhen, China) (37).

#### Data filtering

The raw reads containing more than 50% low-quality bases (quality ≤ 20) or exceeding five ambiguous bases were filtered using FASTP. The remaining reads were aligned to the human genome (Hg19) to eliminate host DNA using bowtie2 (-m 100-600 -v 7 -p 6 -l 30 -r 1 -M 4 -c 0.95). The remaining high-quality reads were utilized to obtain taxonomic and functional profiles.

#### Taxonomic profiling

The MetaPhlAn 3.0 (- input_type fastq - ignore_viruses - nproc 6) was employed to generate profiles of phyla, genera, and species from the high-quality reads derived from metagenomic shotgun sequencing data as previously documented (38).

#### Functional profiling

The HUMAnN 3.0 (-i input_clean_data -o output –threads 10 –memory-use maximum –remove-temp-output) was employed to accurately profile the abundance of microbial metabolic pathways and other molecular functions from metagenomic sequencing data as described (38).

#### Diversity analysis

The alpha diversity was assessed using various indices, including Shannon, Simpson’s, and Inverse Simpson’s indexes, depending on the related taxonomic profiles [R 4.2.1 vegan: diversity (data, index = ‘richness/Shannon/Simpson/InSimpson’)]. Beta diversity was calculated based on the Bray-Curtis distance [(R 4.2.1 ape: pcoa (‘bray_curtis distance’, correction=“none”, rn=NULL)], R 4.2.1 vegan: diversity [data, index = ‘bray_curtis distance’)]. Permutational Multivariate Analysis of Variance (PERMANOVA) was conducted by assessing the abundance profile of gut microbial species/genus through adonis function in R 4.2.1 with 1000 permutations.

### Broad-targeted metabolomic examination and analysis

Broad-targeted metabolomics, also known as untargeted metabolomics, the untargeted metabolomic examination encompasses sample preparation and extraction, UPLC separation, and ESI-Q TRAP-MS/MS detection. The untargeted metabolomics analysis in our study was conducted following previously established protocols.

#### Principal coordinate analysis of microbial and plasma metabolites

PCoA analysis was conducted using the ape comp statistical function in R (version 4.2.1) based on Bray-Curtis distances calculated from the fecal and plasma metabolites profiles.

#### Selection of the differential metabolites between two groups

Differences in microbial composition and plasma metabolites between NDDs children and HCs were assessed using the Wilcoxon rank-sum test. The result was statistically significant at the 0.05 level.

#### Compound annotation and KEGG pathways enrichment analysis

The identified microbial and host blood metabolites of significance were annotated with a KEGG ID by using the KEGG compound database (http://www.kegg.jp/kegg/compound/). The annotated metabolites were subsequently mapped to the KEGG Pathway database (http://www.kegg.jp/kegg/pathway.html).

### Statistical analysis

The differences in the gut microbiota, predicted functional pathways, microbial metabolites, and host plasma metabolites were compared using the Wilcoxon rank sum test. PERMANOVA was employed to assess the impact of various phenotypes on the gut microbial composition at genus and species levels, functionality, fecal and plasma metabolisms. The P value was adjusted using the Benjamini-Hochberg method to control False Discovery Rate (FDR). Associations between significantly different gut microbes and distinctly altered fecal/plasma metabolites were analyzed using Spearman’s rank correlation analysis.

All the statistical analysis were based on packages in R (Version 4.2.1). PERMANOVA: vegan, adonis(t(otu1) ~ phe [,1], data = phe, permutations = 999, na.rm = T). Wilcoxon rank sum test: wilcox.test(as.numeric(pr[i, f1]), as.numeric(pr[i, f2])). Heatmap: pheatmap(cmt,scale = “none”,cluster_row = T, cluster_col = T,display_numbers = pmt). A significance level of P < 0.05 was considered.

## Results

### Phenotypic differences and PERMANOVA analysis of gut microbiota and metabolites

This study included 60 HCs and 40 children with NDDs. Demographic and clinical characteristics showed no significant group differences in sex, age, body mass index (BMI), birth height, birth weight, parental age, maternal childbearing age, delivery mode, or defecation frequency (**Supplementary Table S1A**). However, NDDs children exhibited significant deficits in daily living skills (e.g., independent eating, meal concentration, pre-meal hygiene, tidiness, clothing/toy preferences) and social adaptability (e.g., group activity participation), as assessed by caregiver-reported questionnaires (**Supplementary Table S1A**). Using the Gesell Developmental Diagnosis Scale (GDDS), NDDs children displayed substantial developmental delays: their average developmental month equivalent was 28.63 ± 12.96 months, markedly lower than their chronological age of 5.18 ± 1.77 years. Their mean developmental quotient (DQ) and intelligence quotient (IQ) were 47.02 ± 19.29 and 42.68 ± 17.16, respectively (**Supplementary Table S1A**).

PERMANOVA (Permutational Multivariate Analysis of Variance) revealed significant effects of NDDs status, age, and BMI on both species- and genus-level gut microbiota composition (**Supplementary Table S1L**). At the functional level, NDDs significantly influenced gut microbial pathways annotated by Kyoto Encyclopedia of Genes and Genomes (KEGG). While fecal metabolome profiles showed no direct association with NDDs, age was a significant covariate. For blood metabolites, NDDs status, age, BMI, disease severity, and etiological factors (e.g., icterus, prematurity) all exerted significant effects. To isolate NDDs-related signals, age and BMI were adjusted for in subsequent analyses.

### Characterization of gut microbial composition in NDDs children vs. HCs

**Figure 1:**
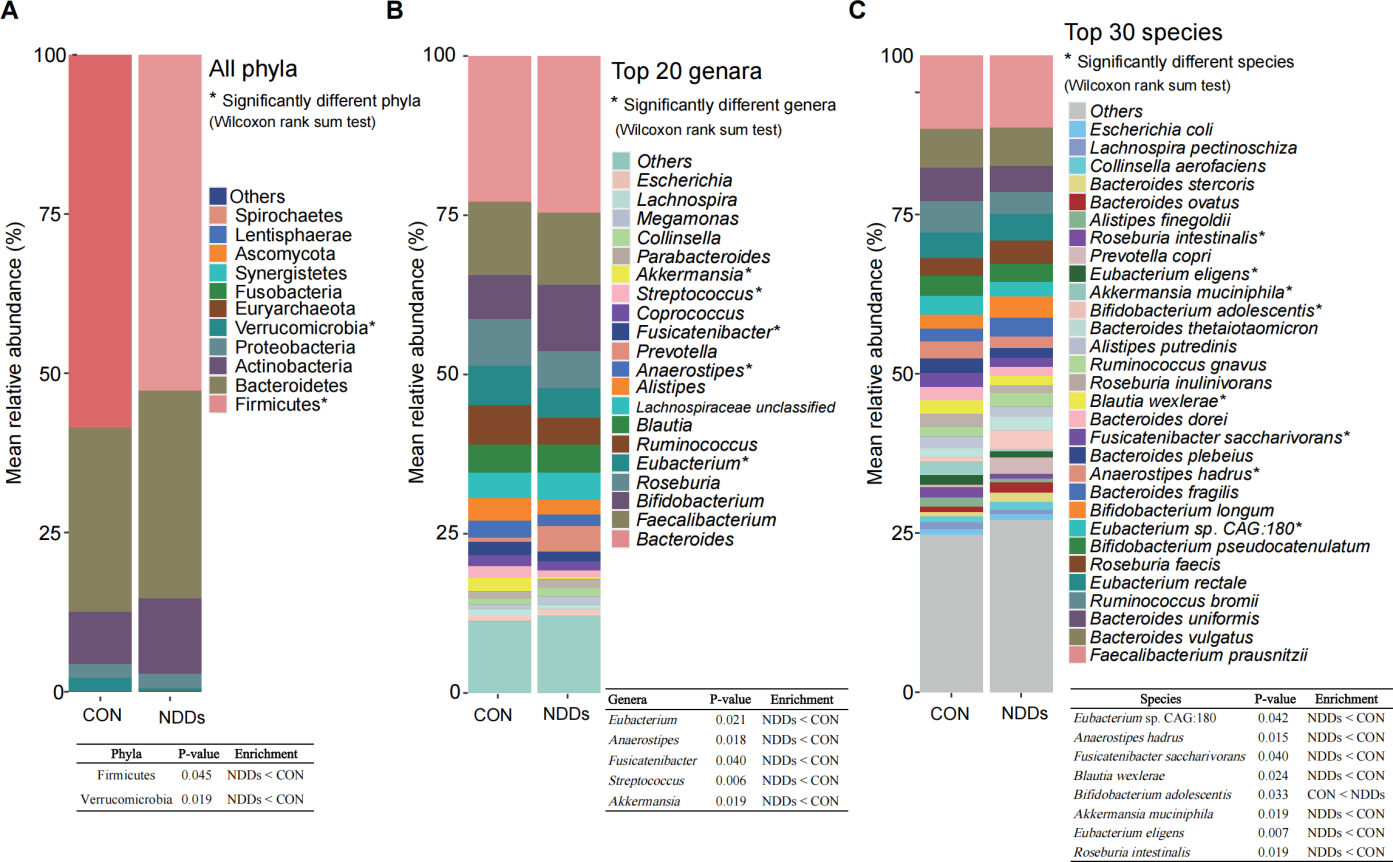
All phyla, top abundant genera and species in the gut microbiota of children with NDDs and healthy controls. **(A)** Distribution of all phyla in both groups. **(B)** The top 20 most abundant genera in both groups. **(C)** The top 30 most abundant species in both groups. *P < 0.05. The table in the figure shows the exact P-values and enrichment directions of significantly different taxa.

Metaphlan 3.0 analysis was employed to identify the taxonomic profiles (**Supplementary Table S1B**). A total of eleven phyla, with Firmicutes, Bacteroidetes, Actinobacteria, Proteobacteria, and Verrucomicrobia comprising the five most abundant phyla across all participants. Notably, Firmicutes and Verrucomicrobia were significantly reduced in NDDs patients compared to HCs (**Figure 1A**, **Supplementary Table S1C**). The Firmicutes/Bacteroidetes (F/B) ratio was 2.03 in HCs versus 1.62 in NDDs patients, reflecting a dysbiotic shift in microbial community structure.

At the genus level, 177 genera were annotated, with the top 20 abundant genera including *Bacteroides*, *Bifidobacterium*, *Faecalibacterium*, *Eubacterium*, and *Roseburia*. Among these, *Eubacterium*, *Anaerostipes*, *Fusicatenibacter*, *Streptococcus*, and *Akkermansia* showed significant decreases in NDDs patients relative to HCs (**Figure 1B**, **Supplementary Table S1D**). At the species level, 522 species were identified, with the top 30 abundant species including *Faecalibacterium prausnitzii*, *Bacteroides uniformis*, and *Eubacterium rectale*. Notably, *Bifidobacterium adolescentis*—a key gamma-aminobutyric acid (GABA)-producing species—was significantly enriched in NDDs patients, while seven species (*Eubacterium* sp. CAG:180, *Anaerostipes hadrus*, *Fusicatenibacter saccharivorans*, *Blautia wexlerae*, *Akkermansia muciniphila*, *Eubacterium eligens*, and *Roseburia intestinalis*) were depleted in NDDs compared to HCs (**Figure 1C**, **Supplementary Table S1E**).

**Figure 2:**
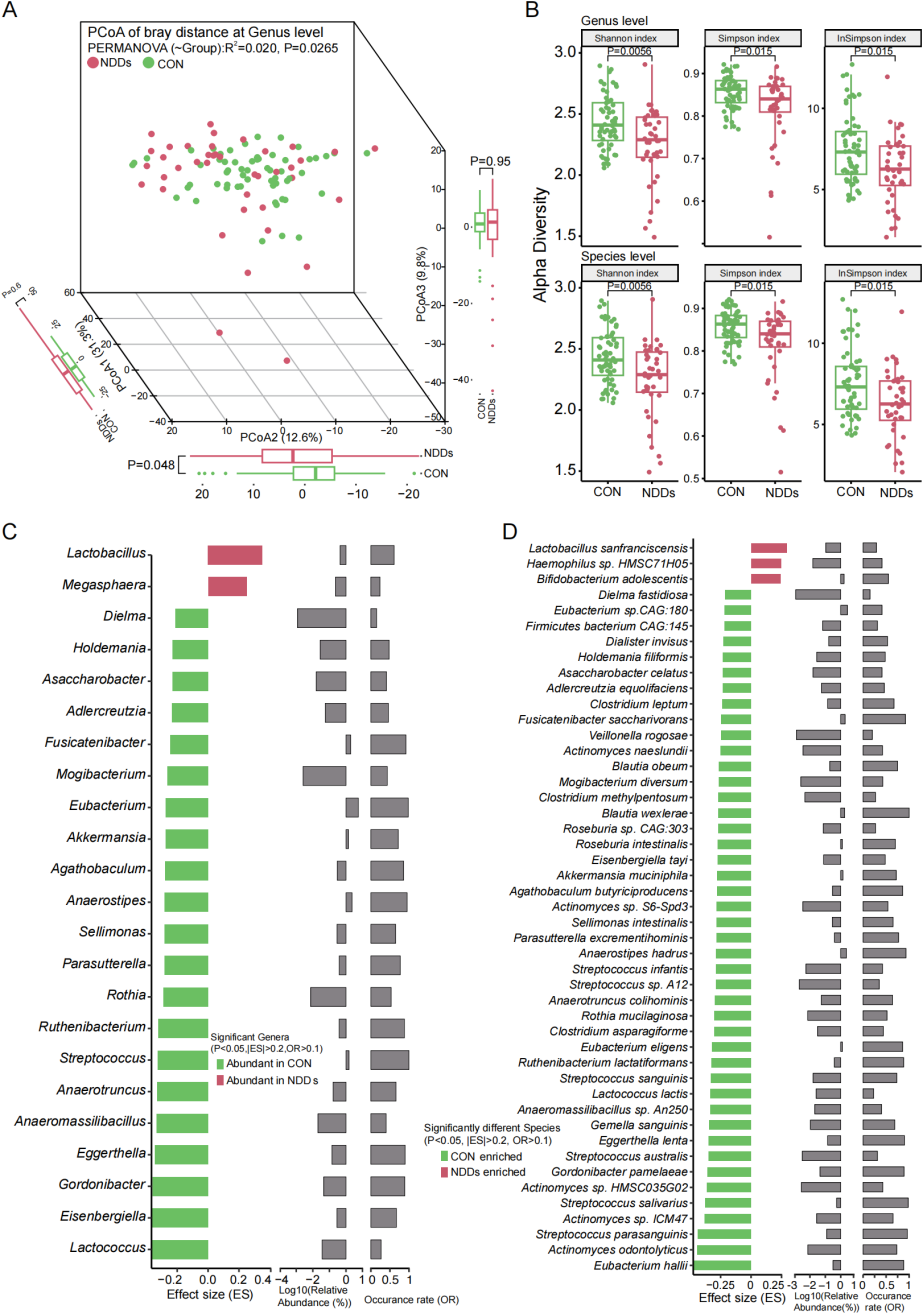
Characteristics and differential analysis of gut microbiota between two groups. **(A)** PCoA based on Bray-curtis distances at the genus level revealed significant compositional divergence between two groups. **(B)** Alpha diversity analysis using Shannon/Simpson/inverse Simpson indices demonstrated significant reductions in gut microbial richness and evenness in children with NDDs at both genus and species levels. **(C)** Significantly different genera between two groups. **(D)** Significantly different species between two groups.

Principal coordinate analysis (PCoA) revealed significant segregation in gut microbial composition between NDDs patients and HCs at both the genus (**Figure 2A**, P = 0.0265) and species (**Supplementary Figure S1**, P = 0.0351) levels. Alpha diversity analyses using the Inverse Simpson, Simpson, and Shannon indices consistently showed reduced microbial diversity in NDDs patients compared to HCs at both taxonomic levels (P < 0.05, **Figure 2B**, **Supplementary Table S1F**). Comparative analysis identified 23 differentially abundant genera and 47 species (P < 0.05, |Effect Size (ES)| > 0.2, Occurrence Rate (OR) > 0.1) between groups. At the genus level, *Lactobacillus* and *Megasphaera* were significantly enriched in NDDs patients, while *Akkermansia*, *Lactococcus*, *Anaeromassilibacillus*, and 18 other genera were depleted (**Figure 2C**). At the species level, *Bifidobacterium adolescentis*, *Lactobacillus sanfranciscensis*, and *Haemophilus* sp. HMSC71H05 were overrepresented in NDDs, whereas *Akkermansia muciniphila*, *Lactococcus lactis*, *Streptococcus salivarius*, and 16 other species were underrepresented (**Figure 2D**). Notably, many NDDs-enriched species, such as *Lactobacillus* spp., are known butyrate producers, suggesting potential links to metabolic dysregulation in NDDs.

### Differences in gut microbial function between children with NDDs and HCs

**Figure 3:**
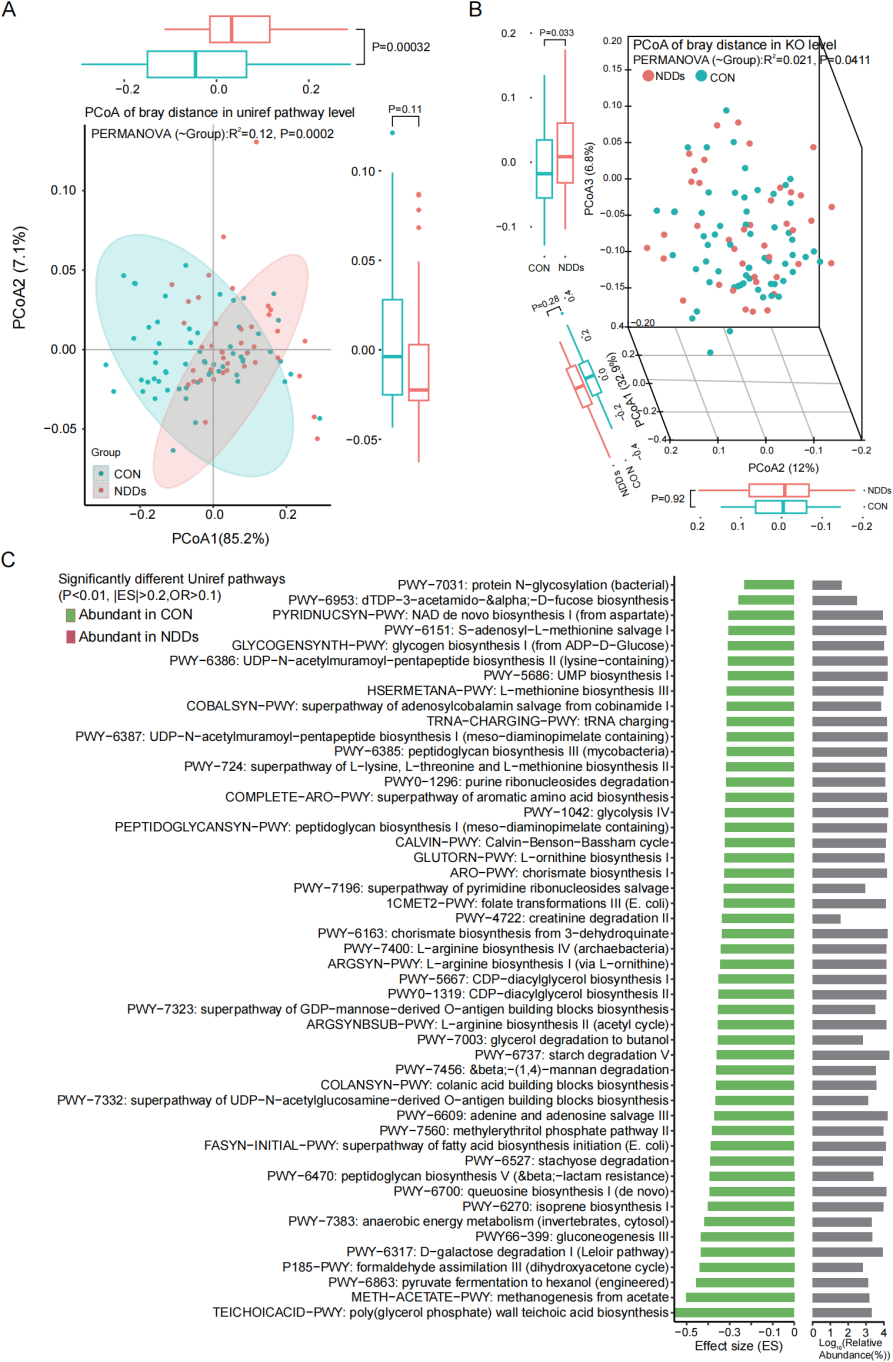
Functional pathway prediction of the gut microbiota. Principal coordinate analysis (PCoA) based on Bray-curtis distances at the Uniref pathways level **(A)** and KEGG Orthology level **(B)** revealed significant differences between two groups. **(C)** The functionally significant pathways were all significantly downregulated in children with NDDs.

HUMAnN 3.0 was used to characterize gut microbial functional profiles (**Supplementary Table S1G**). Principal coordinate analysis (PCoA) based on Bray-Curtis distances revealed significant segregation in microbial molecular functions between NDDs patients and HCs at both the UniProt Reference Clusters (UniRef) pathway level (**Figure 3A**) and KEGG Orthology (KO) level (**Figure 3B**). UniRef pathway analysis identified 452 functional pathways (**Supplementary Table S1H**), of which 49 were significantly differentially abundant (P < 0.01, |ES| > 0.2, Mean OR > 0.1)—all enriched in HCs (**Figure 3C**). These pathways predominantly involved glycometabolism (e.g., glycolysis, stachyose degradation, anaerobic energy metabolism) and amino acid biosynthesis (e.g., L-arginine, L-ornithine, L-methionine).

At the KO level, 4,032 KOs were annotated, with 89 showing significant group differences (P < 0.01, ES > 0.3 or < -0.4, Mean OR > 0.1, **Supplementary Table S1I**). Notably, only eight KOs were enriched in NDDs patients, while 81 were HC-enriched. The latter included enzymes and transporters involved in carbohydrate metabolism (e.g., L-fuculose-phosphate aldolase, ABC-type sugar transport systems), consistent with UniRef pathway findings (**Supplementary Figure S2**).

**Figure 4:**
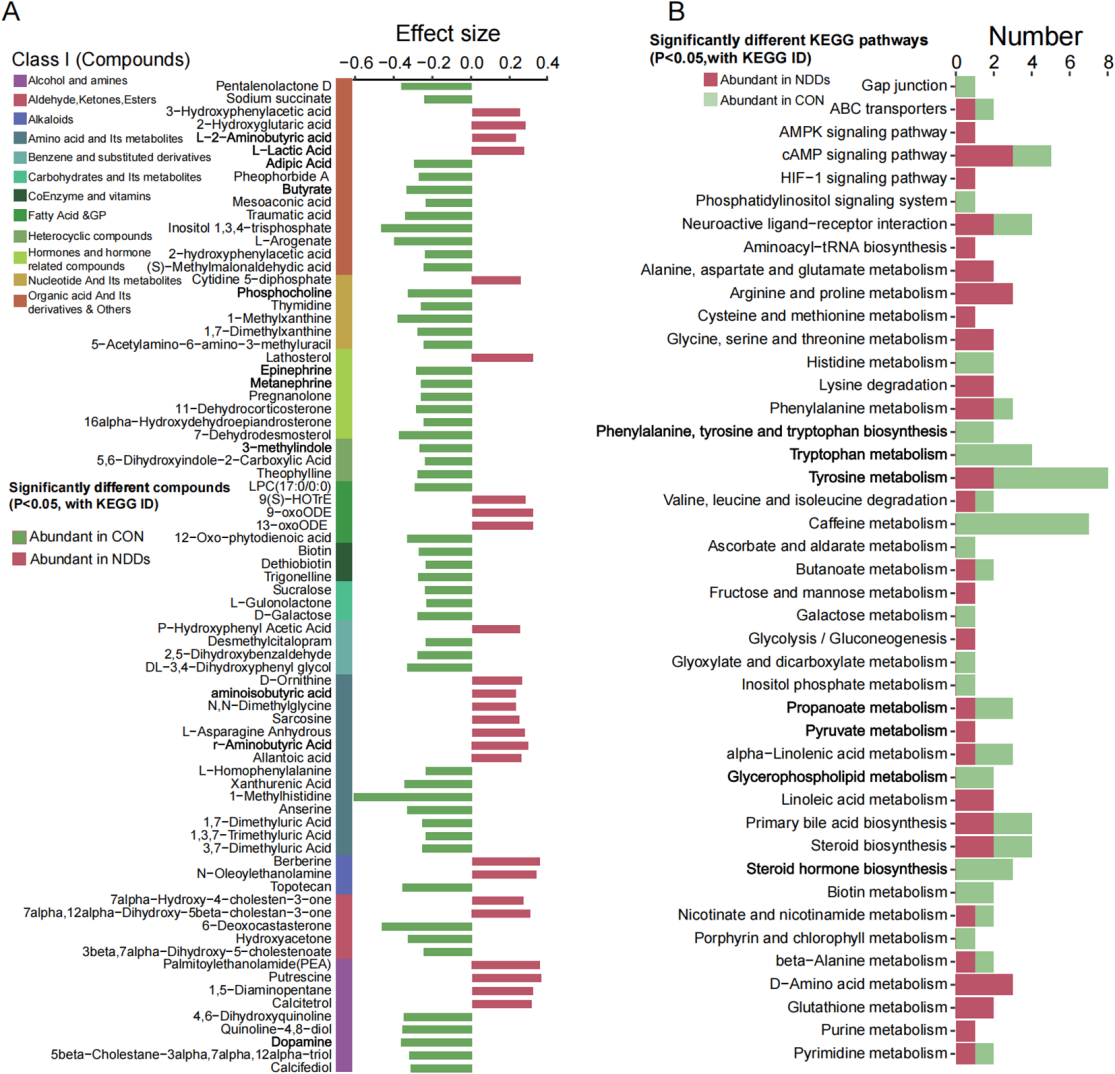
Comparative analysis of fecal metabolism between two groups. **(A)** Significantly different fecal metabolites between two groups. **(B)** KEGG pathways associated with significantly differentially abundant fecal metabolites. (annotated with KEGG IDs, P<0.05; derived from **Figure 4A**).

To further investigate the microbial function, microbial metabolism were detected. A total of 2,689 fecal metabolites were identified, with 44 significantly upregulated in NDDs patients (P < 0.05, FC > 2) and 58 in HCs (P < 0.05, FC < 0.5) (**Supplementary Figure S3**, **Supplementary Table S1J**). NDDs-enriched metabolites included L-2-aminobutyric acid, lactic acid, fatty acid derivatives (e.g., 13-oxoODE, 9-oxoODE), GABA, sarcosine, and putrescine (**Figure 4A**), predominantly mapped to KEGG pathways related to energy metabolism (AMPK, HIF-1 signaling), amino acid metabolism (arginine, proline, glutamate), and lipid metabolism (linoleic acid, glycerophospholipid) (**Figure 4B**).

HC-enriched metabolites comprised short-chain fatty acids (butyrate, propionate), neurotransmitter precursors (tryptophan, phenylalanine, tyrosine), and bioactive molecules (dopamine, epinephrine, 3-methylindole) (**Figure 4A**), linked to pathways such as phenylalanine/tyrosine/tryptophan metabolism, steroid hormone biosynthesis, and phosphatidylinositol signaling (**Figure 4B**). Notably, butyrate, propionate, and monoamine neurotransmitters—key gut-brain axis mediators—were depleted in NDDs, while lactate (implicated in memory formation) showed elevated levels in NDDs feces.

### Alterations in plasma metabolites between NDDs children and HCs

**Figure 5:**
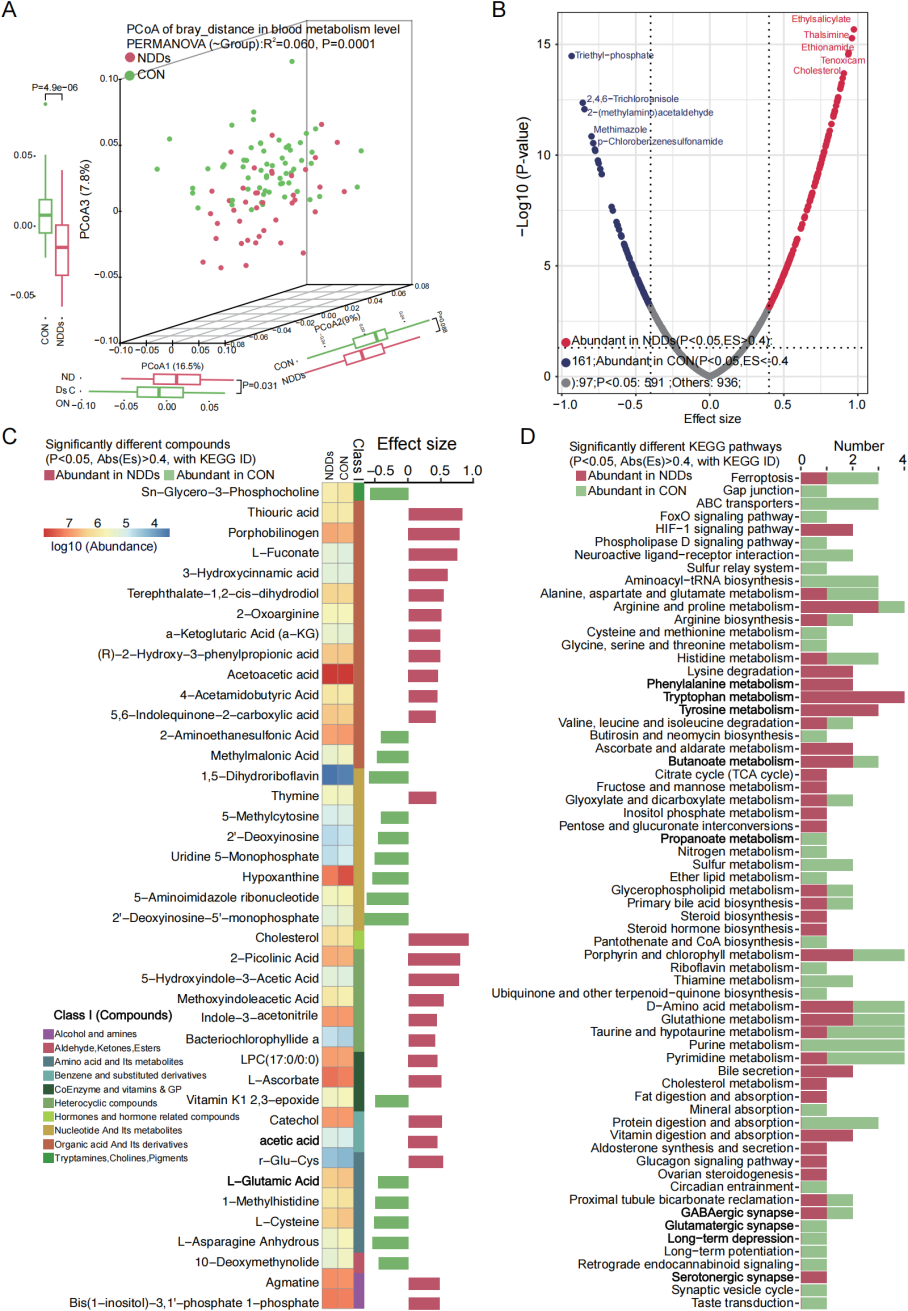
Plasma metabolome profile analysis between two groups. **(A)** PCoA based on Bray-curtis distance of plasma metabolites revealed significant separation between two groups. **(B)** Volcano plot analysis identified markedly different plasma metabolites, with significantly up-regulated (red) and down-regulated (blue) metabolites highlighted. **(C)** Heatmap showed the abundance of significantly different plasma metabolites between two groups. **(D)** KEGG pathways significantly associated with different plasma metabolites.

Plasma metabolomics, which focuses on profiling small-molecule metabolites in blood, offers insights into disease mechanisms and potential therapeutic targets. Using untargeted metabolomics, we identified distinct metabolic signatures in children with NDDs compared to HCs. A total of 1,527 blood metabolites were detected, and principal coordinate analysis (PCoA) based on Bray-Curtis dissimilarity revealed significant separation in metabolic profiles between NDDs patients and HCs (P = 0.0001, **Figure 5A**, **Supplementary Table S1K**).

Differential abundance analysis identified 258 significantly altered metabolites (P < 0.05, |ES| > 0.4), including 161 metabolites enriched in NDDs patients and 97 metabolites enriched in HCs (**Figure 5B**). Mapping these metabolites to the KEGG database annotated 139 metabolites with KEGG IDs, of which 41 were associated with specific metabolic pathways. Among these, 25 NDDs-enriched metabolites—including organic acids, heterocyclic compounds, cholesterol, and acetic acid—were linked to pathways such as arginine/proline metabolism, phenylalanine metabolism, lysine degradation, tryptophan metabolism, tyrosine metabolism, HIF-1 signaling, serotonergic synapse, and TCA cycle. Conversely, 16 HC-enriched metabolites—such as Sn-glycero-3-phosphocholine, 2-aminoethanesulfonic acid, methylmalonic acid, and L-cysteine/L-glutamic acid—were primarily involved in ABC transporters, neuroactive ligand-receptor interaction, glutamatergic synapse, taurine/hypotaurine metabolism, and purine/pyrimidine metabolism (**Figure 5C**).

These findings highlight widespread metabolic dysregulation in NDDs, with perturbations in amino acid metabolism, energy production (TCA cycle), neurotransmitter biosynthesis (serotonergic/glutamatergic pathways), and lipid/cholesterol homeostasis. The enrichment of butanoate and bile secretion pathways in NDDs patients may further link gut microbial metabolism to neurodevelopmental dysfunction, while HC-enriched pathways suggest roles for synaptic signaling and nutrient transport in neurocognitive health.

### Correlation analysis among gut microbiota, fecal, and plasma metabolites

**Figure 6:**
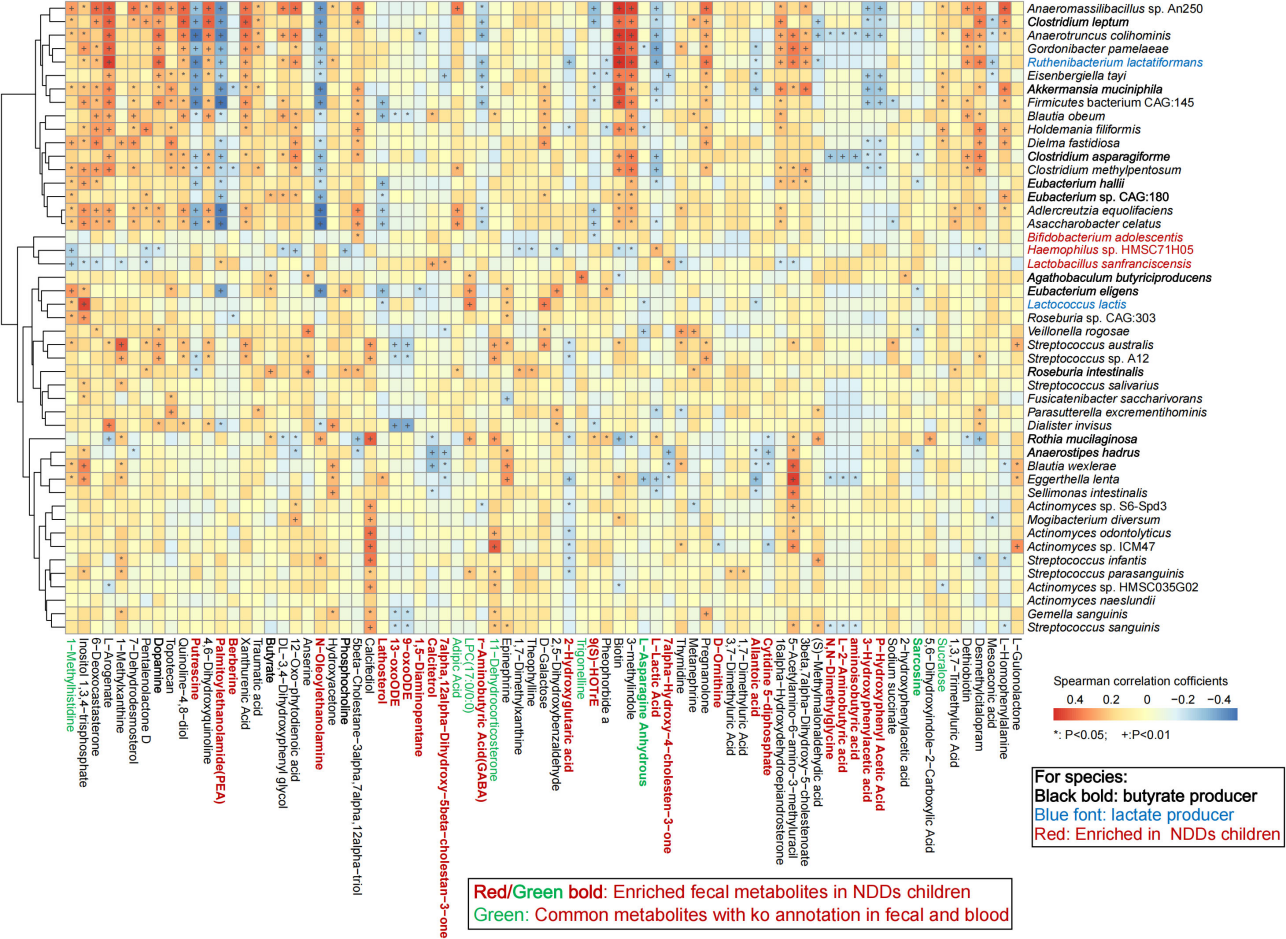
Spearman’s rank correlation analysis revealed the relationships between the significantly different microbial species and the significantly differential fecal metabolites between the two groups. * P < 0.05; + P < 0.01.

To reveal the effect of gut microbiota and metabolites on host metabolism, Spearman’s rank correlation analysis was employed to investigate associations between significantly altered gut microbiota, fecal metabolites, and plasma metabolites (**Supplementary Table S1M**). The analysis revealed a positive correlation between HC-enriched microbial species and HC-specific fecal metabolites, with a reciprocal trend observed in NDDs patients. Notably, *Anaerofustis stercorihominis*, *Clostridium leptum*, *Agathobaculum butyriciproducens*, *Anaerostipes hadrus*, *Eubacterium eligens*, and *Eubacterium hallii*—all depleted in NDDs—exhibited strong positive correlations with fecal butyrate levels (P = 0.0042), which were significantly reduced in NDDs patients. Conversely, lactate-metabolizing microbes including *Megasphaera micronuciformis* and *M. Elsdenii* were enriched in NDDs, while lactate-producing species such as *Clostridium asparagiforme*, *Ruthenibacterium lactatiformans*, and *Lactococcus lactis* were depleted. Fecal GABA—elevated in NDDs with no plasma difference—correlated positively with *B. adolescentis*, an NDDs-enriched species (**Figure 6**, **Supplementary Figure S4**). Fecal dopamine levels—significantly reduced in NDDs—correlated positively with HC-enriched species including *Clostridium leptum*, *Akkermansia muciniphila*, and *Blautia obeum*, but were negatively with NDDs-enriched *Haemophilus* sp. HMSC71H05. Epinephrine—also reduced in NDDs—correlated positively with HC-enriched *Agathobaculum butyriciproducens*, *Eubacterium eligens*, and *Streptococcus sanguinis*. Fecal acetylcholine showed no group difference, but its derivative phosphocholine—positively correlated with *Roseburia intestinalis* and *Eubacterium eligens*—was reduced in NDDs, with a negative correlation to *Haemophilus* sp. HMSC71H05.

At the plasma level, NDDs-enriched species including *B. adolescentis*, *Lactobacillus sanfranciscensis* were correlated with NDDs-specific metabolites such as 4-acetamidobutyric acid and indole-3-acetonitrile (**Supplementary Figure S5**), while HC-enriched species including *Ruminococcus lactatiformans*, *Akkermansia muciniphila* were associated with HC-enriched metabolites like L-glutamic acid and phosphoenolpyruvate. Similar trends were observed for differentially abundant genera versus fecal/plasma metabolites (**Supplementary Figures S6**, **S7**), reinforcing microbiota-metabolism linkages.

**Figure 7:**
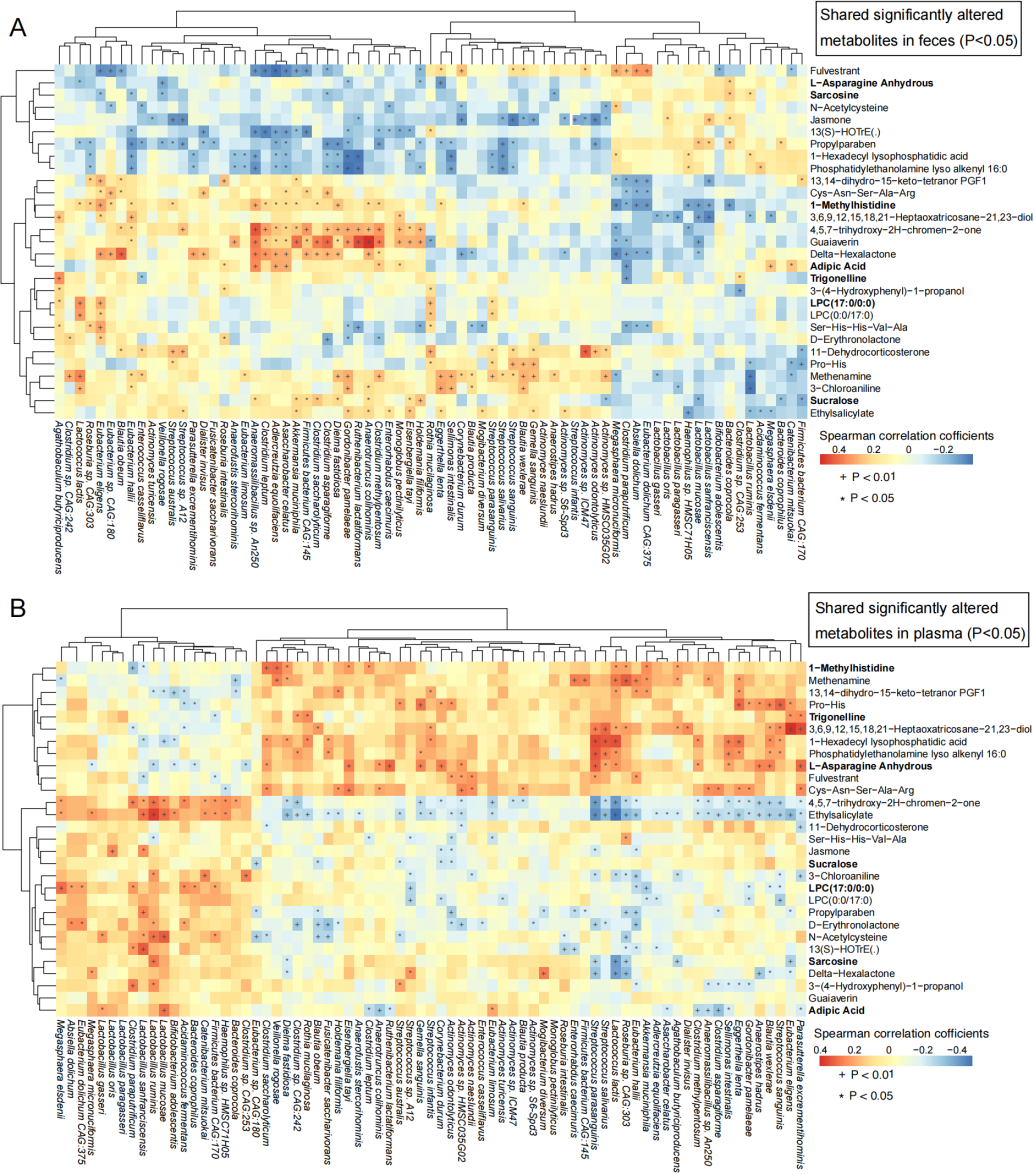
Spearman’s rank correlation analysis between significantly different species and the shared significantly different metabolites in feces and plasma. **(A)** Association study between the gut species and common metabolites from feces. **(B)** Association study between the gut species and common metabolites from plasma. The bold metabolites implicate the metabolites with KEGG map annotated. *P < 0.05; + P < 0.01.

To further explore the comprehensive impact of gut microbiota on intestinal and host metabolism, a Venn analysis was conducted. The analysis identified 29 common differentially expressed metabolites from 354 fecal and 591 plasma metabolites (**Supplementary Figure S8**). Association studies between these metabolites and significantly altered gut microbes showed positive correlations between NDD-enriched gut microbes and NDD-enriched fecal metabolites, while negative correlations were observed between NDD-enriched microbes and HC-enriched fecal metabolites. The same trends were evident in plasma metabolites (**Figure 7**). Among the 29 common metabolites, eight were annotated in KEGG pathways: 11-Dehydrocorticosterone, L-asparagine anhydrous, sarcosine, 1-methylhistidine, LPC(17:0/0:0), trigonelline, adipic acid, and sucralose. Specifically:11-dehydrocorticosterone, LPC(17:0/0:0), adipic acid, and sucralose were decreased in feces but increased in plasma of NDD children; 1-methylhistidine and trigonelline were decreased in both matrices; L-asparagine anhydrous was increased in feces but decreased in plasma; Sarcosine was increased in both (**Figure 7**). These metabolites are primarily involved in steroid hormone biosynthesis, glycine/serine/threonine metabolism, arginine/proline metabolism, choline metabolism in cancer, glycerophospholipid metabolism, and pathways related to amino acid biosynthesis, protein digestion, and mineral absorption. Their enrichment in NDD plasma suggests imbalances in energy and nutrient metabolism in pediatric NDDs.

## Discussion

The gut microbiome and its metabolic activities profoundly influence human neurophysiology and mental health. However, the landscape of gut microbial composition, functional pathways, microbial metabolites, and host blood metabolism in children with NDDs remains poorly defined. Using shotgun metagenomic sequencing and broad-targeted metabolomics, this study systematically characterized differences in gut microbial structure, function, and host-microbial metabolic interactions between NDDs children and age-, sex-, and BMI-matched HCs. Significant disparities were observed in microbial diversity, taxonomic composition, predicted functional pathways, and fecal/plasma metabolites between groups.

### Study cohort and clinical phenotyping

Forty NDDs children and 60 HCs were enrolled, with NDDs patients exhibiting significant deficits in social interaction, self-care abilities (e.g., independent eating, hygiene), and developmental milestones. The average developmental age (28.63 ± 12.96 months) was markedly lower than chronological age (5.18 ± 1.77 years), with developmental quotient (47.02 ± 19.29) and IQ (42.68 ± 17.16) reflecting severe neurodevelopmental impairment. Clinical phenotypes showed no significant group differences in newborn weight/height, parental age, or delivery mode. Notably, children with NDDs were predominantly cared for by grandparents, whereas healthy controls were parent-cared, underscoring caregivers’ critical role during the 0-6-year developmental window. Additionally, NDD children commonly had etiological histories of hyperbilirubinemia, premature birth, or prolonged pregnancy, highlighting the importance of prenatal and neonatal care.

### Gut microbiome dysregulation in pediatric NDDs

Metagenomic analysis uncovered profound differences in gut microbial composition between NDDs patients and healthy controls, characterized by reduced alpha diversity (species richness and evenness) and distinct beta diversity at both genus and species levels. At the phylum level, Firmicutes and Verrucomicrobia were significantly depleted in NDDs, aligning with prior associations of Firmicutes reduction with gastrointestinal and neurobehavioral dysfunction. At lower taxonomic levels, NDDs patients exhibited decreased abundance of 21 genera (e.g., *Eubacterium*, *Akkermansia*, *Lactococcus*) and 44 species, including key short-chain fatty acid (SCFA)-producing taxa such as *Agathobaculum butyriciproducens *(14), *Anaerostipes hadrus* (15), *Clostridium asparagiforme* (16), *Eubacterium hallii* (17), and other beneficial species including *Clostridium leptum* and *Eubacterium eligens. A. butyriciproducens* was reported to improve cognitive impairment in LPS-induced and APP/PS1 mouse models of Alzheimer’s disease (14). *A. hadrus* is a butyrate-producing bacterium capable of metabolizing 5-fluorouracil (15). *C. asparagiforme* produced acetate, lactate, and ethanol as the major products of glucose fermentation (16). *E. hallii* is a butyrate and propionate-producing bacterium from infant feces (17). *E. eligens* can utilize the galacturonide oligosaccharides DP4 and DP5 derived from sugar beet pectin, strongly promotes the production of the anti-inflammatory cytokine IL-10 in *in vitro* cell-based assays (18). *C. leptum* is exceptional inducers of regulatory T cells (Tregs) in the colon and can be considered as therapeutic options for IBD and allergies (19). The decrease of anti-inflammatory and butyrate-producing bacteria in NDDs children reminded us a dysbiosis of the gut microbiota. Consistently, the depletion of these SCFA-producing microbes strongly correlates with reduced fecal butyrate levels in NDDs, consistent with the established role of SCFAs in modulating neuroinflammation and blood-brain barrier integrity in neurological disorders (20). Conversely, NDDs patients showed enrichment of *Lactobacillus*, *Megasphaera*, *Lactobacillus sanfranciscensis*, and *Bifidobacterium adolescentis*. Megasphaera spp., previously linked to gastrointestinal symptoms like abdominal pain and diarrhea in infants (13), may reflect altered gut dysbiosis in NDDs. *Lactobacillus* species, including *L. sanfranciscensis*, a dominant sourdough microbe (21, 22), have been shown to rescue neurobehavioral deficits in preclinical models of maternal microbiome dysbiosis (23), though their functional relevance in NDDs remains unclear. *B. adolescentis*, a known GABA producer (24), was significantly increased in NDDs. This species modulates host metabolism, catalase activity, and lifespan in preclinical models (25), and heat-inactivated strains promote colonic stem cell activation via Paneth-like cells (26). The enrichment of *B. adolescentis* and *L. sanfranciscensis* in NDDs may represent a compensatory host response to microbial dysregulation or dietary influences (e.g., sourdough and buns consumption), though mechanistic validation is needed.

For predicted functional pathways, all the significantly different pathways were highly abudnant in HC group. Further analysis revealed that these microbial pathways were predominantly involved in glycometabolism and amino acid biosynthesis (UniRef/KO analysis), reflecting the metabolic dysfunction of the gut microbiota in patients with NDD. These findings highlight metabolic dysfunction, and a dual pattern of depletion (SCFA producers) and selective enrichment (GABA producers and opportunistic taxa) in the gut microbiota of children with NDDs, underscoring the complex interplay between microbial ecology and neurodevelopmental pathology.

### Metabolic perturbations and gut-brain axis links

Fecal and plasma metabolomics revealed profound metabolic dysregulation in NDDs, with reduced fecal butyrate levels directly linked to depletion of butyrate-producing taxa [such as *Clostridium* spp. and *Eubacterium* spp.). SCFAs are critical mediators of gut-brain signaling and neuroinflammation (27). NDDs patients exhibited reduced abundance of lactate-producing bacteria (*Lactococcus lactis* (28), *Ruthenibacterium lactatiformans* (29) and *Clostridium asparagiforme* (10)] but increased lactate-consuming *Megasphaera micronuciformis* (30), despite undetectable fecal/plasma lactate (likely converted to pyruvate or glucose metabolism). This shift may disrupt lactate’s role in neural excitation and memory formation (31). Elevated fecal GABA in NDDs correlated with *B. adolescentis* (a GABA producer), but reduced plasma GABA derivatives [e.g., glutamate (32)] suggested impaired central nervous system integration. Reduced fecal dopamine and plasma epinephrine in NDDs correlated with HC-enriched microbes further implicated microbial dysbiosis in neurotransmitter deficits (33, 34). Stable fecal acetylcholine levels but reduced phosphocholine (a downstream metabolite) indicated altered cholinergic signaling (35). Tryptophan metabolism revealed reduced fecal kynurenine metabolites (xanthurenic acid, 3-methylindole) alongside increased plasma kynurenine pathway intermediates, reflecting disrupted gut-liver-brain metabolic crosstalk (36). Dysregulated fecal phenolic metabolites (e.g., 3-hydroxy-phenylacetate) and elevated plasma tyrosine derivatives (e.g., thyroxine) highlighted perturbations in phenylalanine/tyrosine metabolism, potentially impacting catecholamine and thyroid hormone biosynthesis.

KEGG pathway annotation indicates significant reprogramming of both microbial and host metabolism in NDDs children. Specifically, in the gut, NDDs children exhibited enhanced amino acid metabolism, accompanied by reduced histidine metabolism and glycerophospholipid metabolism. In contrast, plasma metabolites showed upregulated lipid metabolism, vitamin digestion and absorption, and serotonergic synapse activity, alongside downregulated protein metabolism, glutamatergic synapse function, long-term depression/potentiation, mineral absorption, taurine and hypotaurine metabolism, ABC transporters, and the FoxO signaling pathway. Notably, several pathways displayed opposing trends between gut and plasma: phenylalanine, tyrosine, and tryptophan biosynthesis, as well as tryptophan/tyrosine metabolism and steroid hormone biosynthesis, were reduced in the gut but increased in plasma. Conversely, purine metabolism, cysteine and methionine metabolism, and glycine, serine, and threonine metabolism were enriched in the gut but diminished in plasma. Additionally, three pathways were highly abundant in both gut and plasma of NDDs children: the HIF-1 signaling pathway (which plays a key role in the body’s response to low oxygen concentrations or hypoxia), lysine degradation [via the saccharopine formation and pipecolic acid pathways, clinically linked to severe neurometabolic disorders such as pyridoxine-dependent epilepsy and glutaric aciduria type 1 (39)], and carbohydrate metabolism. Collectively, these results suggest that NDDs children exhibit dysfunction in nutrient and energy metabolism in both the gut and plasma, with abnormal redox reactions potentially contributing to these metabolic perturbations.

Correlation analysis between significantly differential species and fecal/plasma metabolites revealed several insightful interaction networks that merit consideration in future studies. Butyrate, whichxwas significantly decreased in the gut of children with NDDs, showed a significant positive association with *Rothia mucilaginosa*, *Roseburia intestinalis*, *Eubacterium eligens*, *Agathobaculum butyriciproducens*, and *Eubacterium* sp. CAG:180; and also exhibited positive association with known butyrate-producers including *Anaerostipes hadrus* and *Eubacterium hallii*. Dopamine, another metabolite significantly reduced in NDDs children, was significantly positively correlated with NDDs-downregulated species including *Clostridium leptum*, *Anaerotruncus colihominis*, *Gordonibacter pamelaeae*, *Ruthenibacterium lactatiformans*, *Eisenbergiella tayi*, *Akkermansia muciniphila.* Phosphocholine, which was significantly decreased in NDDs children, showed a significant positive association with *Eubacterium eligens* and *Roseburia intestinalis*, but a negative association with *Haemophilus* sp. HMSC71H05. These results suggest that supplementation with butyrate, dopamine, and choline may increase the relative abundance of these beneficial species (which are reduced in NDDs children) and thereby alleviate NDDs symptoms. Conversely, L-lactic acid and GABA, which were significantly elevated in children with NDDs, displayed a significant negative correlation with beneficial species enriched in healthy controls (HCs) while showing a positive association with NDDs-enriched *Haemophilus* sp. HMSC71H05. This implies that reducing the abundance of lactate- and GABA-producing species could also mitigate the clinical symptoms of NDDs.

While this study establishes a foundation link between gut microbiome-metabolome interactions and NDDs in children, it has certain limitations that warrant attention in future research, including the use of a single-center cohort and the lack of mechanistic validation. In subsequent studies we aim to expand the sample size and enhance geographic diversity to account for regional variations in the microbiota. We will also utilize animal models to dissect causality between specific microbes/metabolites and NDDs phenotypes. Additionally, the effects of metabolites on the growth of specific microorganisms should be investigated, and microbiota-targeted interventions—such as probiotic supplementation and short-chain fatty acid (SCFA) administration—will be explored for their therapeutic potential in pediatric NDDs.

In conclusion, this study systematically characterized the gut microbiome, microbial and host metabolome profiles in pediatric NDDs, underscoring complex microbiota-host metabolic interactions, identifying dysregulated SCFA/lactate-producing bacteria, neurotransmitter deficits, and aromatic amino acid metabolism abnormalities, as well as metabolic disturbances of major energy and nutrient metabolism including carbohydrates/proteins/fat digestion and absorption. Our findings provide a novel framework for understanding gut-brain axis involvement in pediatric NDDs and prioritize microbial-metabolite targets for diagnostic and therapeutic development.

## Data Availability

The relevant data have been deposited in the China National GeneBank Database (CNGBdb) under the accession number CNP0004326.

## Supplementary material

Supplementary Table 1
**(A)**. Phenotypes of the subjects. **(B)**. Taxonomic profiling of the gut microbiota. **(C)**. Phyla analysis. **(D)**. Genera analysis. **(E)**. Species analysis. **(F)**. Diversity calculation of the gut microbiota at phyla, genera, and species levels. **(G)**. Functional analysis of the gut microbiota. **(H)**. Predicted functional pathways of the gut microbiota. **(I)**. KO analysis of the gut microbiota. **(J)**. Fecal metabolites analysis of the gut microbiota. **(K)**. Plasma metabolites analysis of the gut microbiota. **(L)**. PERMANOVA of various phenotypes on gut microbial composition, function, fecal and blood metabolites. **(M)**. Spearman’s rank correlation analysis between the significantly different species and fecal/plasma metabolites.
